# Prolonged lumbosacral pain as the initial presentation in acute lymphoblastic leukemia in an adult: A case report

**DOI:** 10.1097/MD.0000000000015912

**Published:** 2019-06-14

**Authors:** Fanglin Li, Jinxia Wang, Aifei Liu, Liuyan Xin, Sisi Zhong, Yang Hong, Yijian Chen

**Affiliations:** a Hematology Department; b Blood Transfusion Department, The First Affiliated Hospital of Gannan Medical University, Ganzhou, Jiangxi Province, PR China.

**Keywords:** acute lymphoblastic leukemia, case report, lumbosacral pain, MLL-AF4 fusion protein

## Abstract

**Rationale:**

The differential diagnosis of conditions manifesting as bone and joint pain is complex. Although many individuals with acute leukemia experience bone pain, lumbosacral pain as an early feature of acute lymphoblastic leukemia (ALL) is rare.

**Patient concerns:**

Here we report a case of an adult who presented with a 7-month history of persistent lumbosacral pain which had become more severe during the previous month.

**Diagnoses:**

Prior to referral, his full blood count revealed no abnormalities, and a computerized tomography scan revealed mild bone hyperplasia of his lumbar vertebrae, with disc herniations of L3–S1. His blood biochemistry and urinary test results had been normal. After referral to our clinic, tests of the morphology, immunology, cytogenetics, and molecular biology of his bone marrow led to a diagnosis of MLL-AF4 fusion positive B-cell ALL.

**Interventions:**

Prior to his referral, he had been treated with painkillers by local doctors. The painkillers initially provided pain relief, but their effect wore off over time. After diagnosis, he was started on an adult ALL chemotherapy protocol.

**Outcomes:**

His symptoms resolved within a week of starting chemotherapy. At his most recent assessment, 10 months after diagnosis, he was on maintenance chemotherapy and in remission.

**Lessons:**

This case illustrates that prolonged lumbosacral pain may be a symptom of a life-threatening condition, rather than only attributable to chronic inflammation or disk herniations. Therefore, clinicians need to pay attention to subtle differences in the clinical presentation of patients with lumbosacral pain.

## 1 Introduction

Although lumbosacral pain is quite common in the general population, it is also a common feature of many diseases, including rheumatic disease, disc herniations, spinal neoplasms, and spinal infections. Because of this, the diagnosis of conditions manifesting with lumbosacral pain is difficult. According to one report, leg pain is present in up to 57.5% of all cases of low back pain.^[1]^ Back pain and leg pain are both non-specific symptoms, so they are of limited help in making a clinical diagnosis.

Acute Lymphoblastic Leukemia (ALL), a malignant hematologic disease, is reported to be the most common malignancy in children, but is less common in adults.^[2]^ Adults with ALL chiefly present with anemia, bleeding, infections, pallor, lymphadenectasis, and bone pain. Back pain has been reported as an early manifestation of ALL in children^[3]^ but, to the best of our knowledge, there have not been any previous reports of chronic back pain as an initial manifestation of ALL in adults.

Here we report on a case of a man with ALL, who initially presented with prolonged lumbosacral pain. Our intention in writing this case report is to make other clinicians aware that ALL in adults can present as lumbosacral pain without other systemic symptoms. This case study was approved by the Biomedical Research and Ethics Committee of The First Affiliated Hospital of Gannan Medical University, and the patient has provided written informed consent for the publication of this case report.

## 2 Case presentation

A 30-year-old man presented with a 7-month history of mild lower back pain. He had no history of trauma, morning stiffness, oral ulcers, fever, hair loss, erythrasma, or weight loss. He had been treated by local doctors, but they had failed to make a diagnosis. Treatment had initially provided pain relief, but his lumbosacral pain had recurred.

A month before he was seen at the rheumatology outpatient clinic in our hospital, his lumbosacral pain had become more severe, and he had developed pain in both his legs and his knees, which was more severe on the right. The pain had been severe enough to restrict his daily activities and was present at night, disturbing his sleep. His other symptoms included a dry mouth. He was treated by local doctors again, but this time he did not respond to treatment. A localized computerized tomography (CT) scan revealed mild bone hyperplasia of the lumbar vertebrae and disc herniations in L3–S1, prompting referral to the rheumatology department of our hospital. On physical examination, he was found to have percussion pain over his lumbosacral region and restriction of movement when doing squats. Examination of other systems revealed no abnormalities. The rheumatologists made a provisional diagnosis of spinal arthritis.

**Figure 1:**
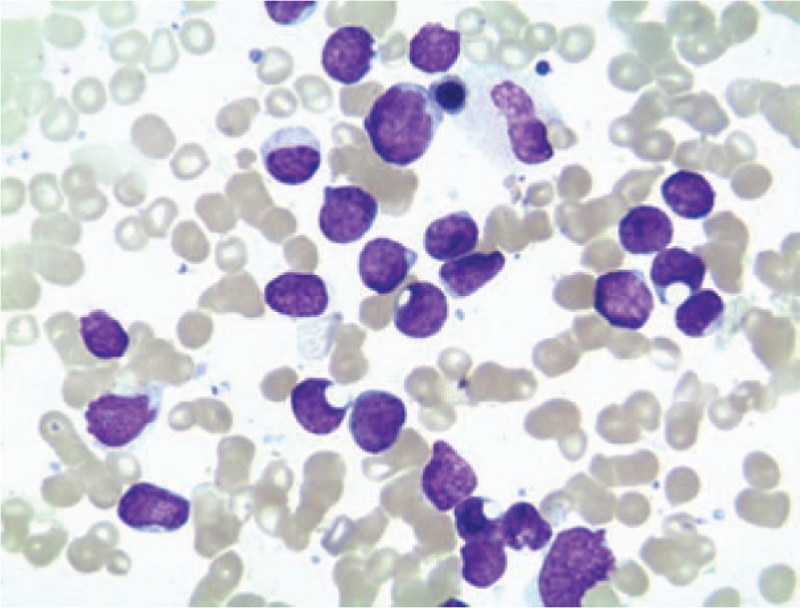
Bone marrow smear showing blast lymphocytes. The blast lymphocytes are round, elliptic, or irregular in shape, and the size of the cells is varied, with large lymphocytes predominating.

A peripheral blood test found a white blood cell count of 3.48 × 10^9^/L (normal range, 3.5–9.5), red blood cell count of 3.87 × 10^9^/L (normal range, 4.3–5.8), hemoglobin level of 11.4 g/dL (normal range, 13–17.5), platelet count of 215 × 10^9^/L (normal range, 125–350), and 48.3% lymphocytes (normal range, 20–50). His C-reactive protein level was 2.29 mg/dL (normal range, <0.3) and his rheumatoid factor level was 12 U/mL (normal range, <25). Tests for antinuclear antibody, anti-cyclic citrullinated peptide, antikeratin antibody, and antineutrophil cytoplasmic antibodies (A-ANCA, C-ANCA, and P-ANCA) were all negative. His chest X-ray was normal. Magnetic resonance imaging of his sacroiliac area revealed inverse transformation of the bone marrow, prompting his clinicians to do a bone marrow biopsy. This revealed 84% blast cells and B-cell lymphoblastic leukemia (Fig. 1).

**Figure 2:**
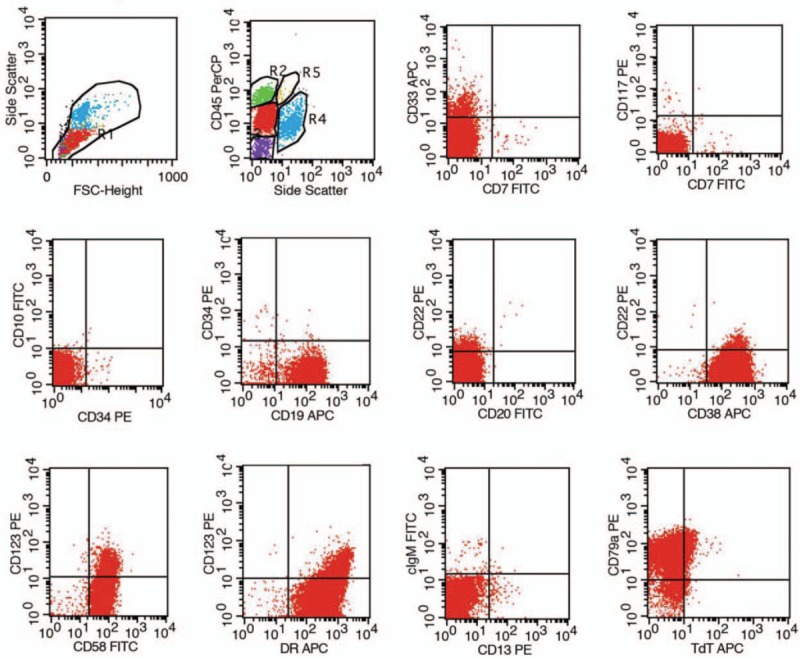
Immunophenotyping of a bone marrow sample. Lymphocytes were characterized by a typical B-ALL immunophenotype: CD79a+ (94.18%), CD19+ (98.19%), CD5+ (99.32%), CD38+ (99.51%), HLA-DR+ (99.45%) and negative for CD3 and MPO.

**Figure 3:**
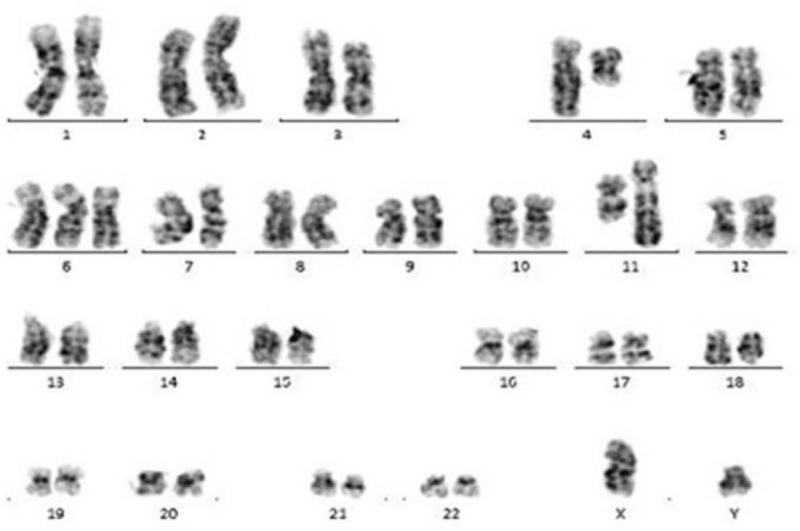
Cytogenetic analysis of a bone marrow sample showing an abnormal karyotype: 47, XY, t(4; 11)(q21; q24), +6(4)/46, XY(16).

**Figure 4:**
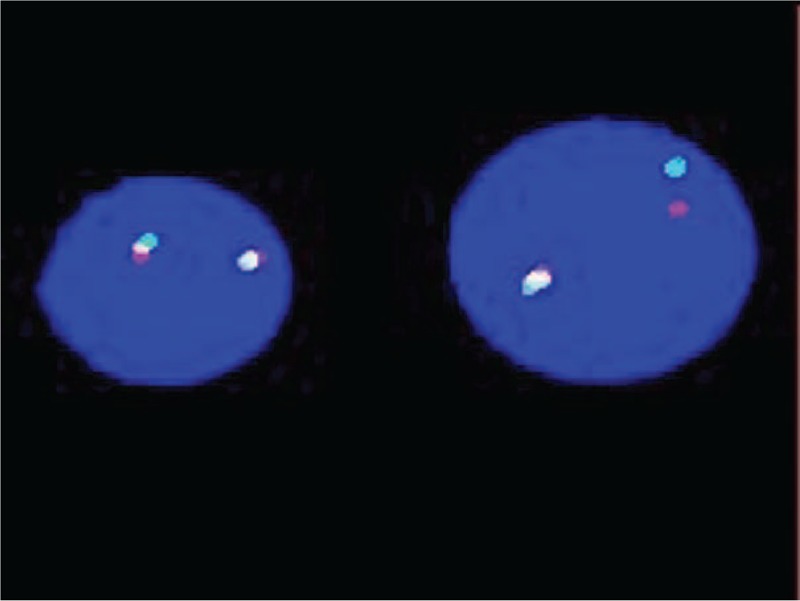
Fluorescence in situ hybridization on a bone marrow sample showing nucish (MLL×2)(5’MLL sep 3’MLL×1).

After providing informed consent to referral, he was referred to our hematology department for further investigations. Immunophenotypic analysis, performed on bone marrow aspirate samples, confirmed the previous findings. The diagnosis of ALL was confirmed by demonstration of expression of the B-cell markers: CD19, HLA-DR, CD58, CD79a, TDT, CD123, and CD38 (Fig. 2). Traditional cytogenetic analysis was performed on interphase cells prepared from bone marrow samples cultured for 24 hours without mitogens, using standard technology.^[4,5]^ The result was reported as an abnormal karyotype 47,XY,t(4;11)(q21;q24),+6(4)/46,XY(16) (Fig. 3). Fluorescence in situ hybridization analysis was performed to test for common abnormalities associated with ALL, according to the manufacturer's instructions. The results were positive for MLL-AF4 (Fig. 4), which is present in 6% of adults with ALL, and indicates a poor prognosis.^[6,7]^ He was started on an adult ALL chemotherapy protocol. His back pain resolved within a week of starting chemotherapy. At his most recent assessment, 10 months after diagnosis, he was on maintenance chemotherapy and in remission (Table 1).

**Table 1:**
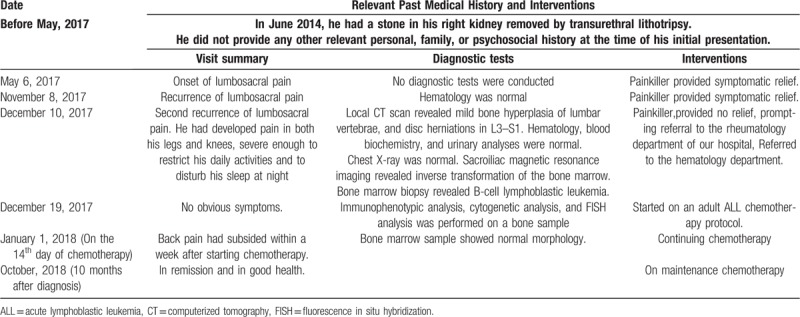
Timeline of clinical assessments, diagnostic tests, and treatment of the case discussed in this case report.

## 3 Discussion

ALL is one of the most common forms of acute leukemia in adults, accounting for 20% to 30% of cases. Different study groups currently use relatively uniform diagnostic criteria and systematic treatment protocols. One study found a complete remission rate of 70% to 90%, and a 3 to 5-year disease-free survival of 30% to 60%.^[7]^

The clinical symptoms of ALL are variable. Adults with ALL most commonly present with anemia, bleeding, infections, pallor, lymphadenectasis, and bone pain. Less common manifestations include visual abnormalities, osteoporosis, parotidomegaly, breast lumps, pericoronitis, arthritis, bone lesions, generalized osteopenia.^[8–13]^ In adults, lumbosacral pain is an unusual presentation of leukemia. However, leukemia can cause significant lumbosacral pain in the absence of other systemic symptoms. Because there are no characteristic symptoms of ALL, and lumbosacral pain is very common, the diagnosis of ALL in adults presenting with lumbosacral pain can be difficult and diagnosis is usually delayed.^[14]^ When classic features of ALL such as anemia, high or low white blood cell counts with lymphocytosis, thrombocytopenia, and a clear picture of bone marrow involvement are present, it is easier to make a diagnosis.

It is difficult to determine how often patients with an undiagnosed ALL present to clinicians with lumbosacral pain because there have been few cases reported to date. Most reports of ALL presenting as lumbosacral pain have been in children, and there have been no previous case reports of ALL presenting as lumbosacral pain in adults

Bone pain is one of the initial manifestations of ALL that may occur as a result of direct leukemic infiltration of the periosteum, bone infarction, or expansion of the marrow cavity by leukemic cells.^[15]^ About two-thirds of children with ALL will have had signs and symptoms of disease for less than 4 weeks at the time of their diagnosis, a history of signs and symptoms for several months does not rule out a diagnosis.^[16]^ Adults with ALL may also experience bone pain for several months prior to diagnosis, as is illustrated by this case.

Lumbosacral pain is a fairly common health problem and is reported to be one of the chief causes of activity limitation and work absence in the world.^[4,17]^ It is not surprising, therefore, that lumbosacral pain is the most common reason for people consulting chiropractors.^[18]^ The most common acquired causes of lumbosacral pain in adults are trauma, inflammation, muscle strain, disc herniations, and tumors. Patients who experience recurrent lumbosacral pain, should be given a thorough diagnostic work-up.

The patient who is the subject of this report, suffered a lot, and it took a total of 7 months from the onset of his lumbosacral pain to the diagnosis of ALL. During this period his lumbosacral pain was recurrent and became progressively more severe. Initially, there were no significant abnormalities on hematology, blood biochemistry or X-ray, CT scan imaging. Early diagnosis, and early treatment are key to achieving the best possible outcomes for patients with ALL. After the patient was diagnosed, he was started on an adult ALL chemotherapy protocol. He responded well to chemotherapy, and at his most recent assessment, he was still in remission.

## 4 Conclusions

It is important to be aware that ALL can present as lumbosacral pain. Clinicians need to pay attention to minor changes in the clinical presentation of patients with lumbosacral pain. Prompt referral to specialists, and cooperation between clinicians, is crucial to ensure the correct diagnosis of ALL. Only in this way can clinicians avoid unnecessary diagnostic procedures, prevent rapid progression of the disease, and improve patient outcomes.

## Acknowledgments

We thank all the staff of the hematology, histopathology, cytogenetic and molecular laboratories for collecting the data and for their technical help.

## Author contributions

FL: conception and design, data collection, drafting of the manuscript; JW, AL, LX, SZ, and YH: data collection, drafting of the manuscript; YC: conception and design, drafting of the manuscript. All authors read and approved the final manuscript.

**Conceptualization:** Yijian Chen.

**Funding acquisition:** Yijian Chen.

**Resources:** Fanglin Li, Jinxia Wang, Aifei Liu, Liuyan Xin, Sisi Zhong, Yang Hong.

**Validation:** Yijian Chen.

**Writing – original draft:** Fanglin Li, Jinxia Wang, Aifei Liu, Liuyan Xin, Sisi Zhong, Yang Hong, Yijian Chen.

**Writing – review & editing:** Yijian Chen.
